# Induced Pluripotent Stem Cells (iPSCs) and Gene Therapy: A New Era for the Treatment of Neurological Diseases

**DOI:** 10.3390/ijms222413674

**Published:** 2021-12-20

**Authors:** Giulia Paolini Sguazzi, Valentina Muto, Marco Tartaglia, Enrico Bertini, Claudia Compagnucci

**Affiliations:** Genetics and Rare Diseases Research Division, Bambino Gesù Children’s Hospital, IRCCS, 00146 Rome, Italy; giulia.paolini@outlook.it (G.P.S.); valentina.muto@opbg.net (V.M.); marco.tartaglia@opbg.net (M.T.); enricosilvio.bertini@opbg.net (E.B.)

**Keywords:** stem cells, gene therapy, iPSCs, viral vector, non-viral vector, neurodegeneration, pediatric diseases, CRISPR-Cas9 gene editing

## Abstract

To date, gene therapy has employed viral vectors to deliver therapeutic genes. However, recent progress in molecular and cell biology has revolutionized the field of stem cells and gene therapy. A few years ago, clinical trials started using stem cell replacement therapy, and the induced pluripotent stem cells (iPSCs) technology combined with CRISPR-Cas9 gene editing has launched a new era in gene therapy for the treatment of neurological disorders. Here, we summarize the latest findings in this research field and discuss their clinical applications, emphasizing the relevance of recent studies in the development of innovative stem cell and gene editing therapeutic approaches. Even though tumorigenicity and immunogenicity are existing hurdles, we report how recent progress has tackled them, making engineered stem cell transplantation therapy a realistic option.

## 1. Introduction

Nearly ten years have passed since the Nobel prize was awarded to Shinya Yamanaka for having successfully reprogrammed somatic cells into pluripotent stem cells [1,2]. By introducing four genetic transcription factors (*Oct 3/4, Sox-2, c-Myc, Klf 4*) in fibroblasts, his team observed an endophenotype recapitulating the embryonic stem cell status, establishing the induced pluripotent stem cells (iPSCs) technology [1,2]. Currently, iPSCs are used as in vitro models to explore the pathophysiological mechanisms in human diseases, which comes particularly useful to researchers to perform mechanistic studies using the relevant cellular context of disease and, eventually, therapy strategies [3,4,5]. Within this framework, iPSCs represent an indefinite source of patient-derived cells [5]. iPSC technology resulted particularly useful for understanding the pathophysiology of human neurological disorders difficult to study because of the inaccessibility of the tissue of interest, as the nervous system. In addition to this, the therapeutic approaches for tissues other than the nervous system have an established history. Meanwhile, on the contrary, gene therapy approaches targeting the central nervous system are very recent and for this reason we will focus on iPSCs and gene therapy in neurological disorders.

Within the last decades, extensive efforts have been spent on discovering more effective and more tolerable methods of delivering gene therapy [6]. Most relevant work has been focused on selectively targeting therapeutic genes by achieving their permanent transduction and by minimizing cytotoxic effects with the use of viral vectors. Moreover, the recent development of on-edge genome editing techniques, including the CRISPR-Cas9 technology and transcription-activator like effector nucleases (TALENs), has opened the road for innovative therapeutics [7,8]. These methods allow for the restoration of deficient proteins, correction of mutations in situ, removal detrimental mutations, and addition of genes at precise genome sites [7]. Moreover, CRISPR-Cas9 gene editing applied to the iPSC technology increases the chance of obtaining isogenic mutated lines and allows for gene correction in iPSCs aimed at the therapeutic rescue of genetic diseases. Advances in the CRISPR-Cas9 technology have boosted its efficiency by guiding the delivering of the nuclease system. An extracellular nanovesicle-based ribonucleoprotein delivery system known as NanoMEDIC was recently built to effectively induce permanent exon skipping for in vivo genome editing [9]. Similarly, a CRISPR Guide Assisted Reduction of Damage (CRISPR GUARD) co-delivering short RNAs to guide the CRISPR-Cas9 system, hence minimizing off-target mutagenesis, has been recently developed [10]. These latest advances together with the iPSC technology have set the stage for a permanent and efficient treatment for neurological disorders.

The incidence of neurological disorders has largely risen as the world population is becoming elder. However, these diseases represent a social burden also in the childhood population. Pediatric neurological diseases include a large and emerging number of clinically heterogeneous neurodevelopmental and neurodegenerative conditions, including ataxias, muscular dystrophies, lysosomal and mitochondrial disorders, spinal muscular atrophy (SMA), and congenital retinal degenerative pathologies. Sadly, no efficient pharmacological treatment is known. To this aim, gene therapy has the potential to represent an effective and permanent cure [11,12]. However, replacing damaged cells with engineered patient-specific iPSCs able to integrate permanently and specifically in the proper “niche” represents the Next Generation Gene Therapy, hopefully to be considered as the gold standard therapy in the near future.

Ever since their first findings, the work performed by Yamanaka and coworkers has focused many research efforts on stem cells, particularly on retinal degeneration diseases, but also on other neurological disorders [13]. These preliminary efforts forge a path for cell replacement therapy as the newest and most effective gene therapy [14]. It is now largely accepted in the scientific community that stem cell therapy offers a unique therapeutic approach for several untreatable human diseases, although some relevant issues must be taken into consideration [15]. These include critical concerns related to tumorigenicity, heterogeneity, and immunogenicity [15]. In the present review, we discuss the potential of stem cell therapy compared to classical gene therapy by overviewing the currently used methods. While classical gene therapy mainly uses viral vectors to transfer genes, stem cell replacement gene therapy uses advanced methods such as CRISPR-Cas9 to correct genes and later introduce engineered iPSCs in patients. Hence, we consider the latest findings on gene therapy tested on iPSCs models and, most importantly, we illustrate the newest insights coming from the stem cell research and clinical centers. We will address the question whether the iPSCs technology and its applications have the potential to open a new future in treating neurological disorders, with a special focus on pediatric diseases.

## 2. Gene Therapy so Far

With the growing knowledge in genetics and genetic engineering, scientists came up with the idea that genes could be used as therapeutics to restore proper function in affected cells [16,17]. Gene therapy started in the United States at the end of the 1980s with the oncologist Steven Rosenberg who traced reinfused T lymphocytes with a retroviral vector containing a genetic marker [17,18,19]. Earlier, Rogers and Pfuderer brought the first proof of concept that viral RNA/DNA could be transduced and used to transfer genetic material [18,19,20]. In 2003, the first gene therapy drug was approved in China under the name of Genidicine [21], an adenoviral vector used to treat squamous cell carcinoma with very small side effects [21]. The approval of this drug opened the world of gene therapy. In Europe, the first gene therapy drug recommended for commercial approval named Gylbera, was an adenoviral vector used for restoring lipoprotein lipase expression for the treatment of lipoprotein lipase (LPL) deficiency [22].

In the beginning, gene therapy was mainly exploited to treat monogenic recessive diseases [17], most of the time with disappointing results [23]. Recently, several gene therapy products are coming on the way for approval for the treatment of neurodegenerative disorders, haemophilia, immune diseases, cancer, and eye-degenerative diseases [6]. The second decade of gene therapy has now a more robust potential and looks more feasible [24]. Yet, the third decade will open shortly, and we anticipate that replacement stem cell therapy will play a major role in opening the next era of gene therapy.

### 2.1. Classical Delivery Methods

Classical delivery methods commonly imply gene transfer through viral vectors and non-viral vectors aimed at restoring defective genes. Below, we will briefly discuss the most commonly used delivery methods.

#### 2.1.1. Viral Vectors

The most common method used until now to deliver therapeutic genes into cells uses viral vectors due to their biological nature of integrating their genome into the hosts [8,17]. Gene therapy delivered with viral vectors relies on engineered viruses with the deliverable genetic material and no pathogenicity [8]. The efficiency of viral vectors also relies on the strong specificity for cell types [8]. Currently, the main clinically used viral vectors include retroviruses, lentiviruses, adenoviruses, adeno-associated viruses (AAV), and herpes viruses [8,17,25,26]. The genome of retroviruses and lentiviruses consists in single-stranded RNA. Infection requires conversion of their genome into DNA and integration in the hosts’ genome. Interestingly, lentiviruses can also infect quiescent cells, making their use particularly relevant in gene therapy [17]. On the other hand, adenoviruses are double-stranded DNA that do not integrate in the host genome but are known to strongly activate the immune system [27]. AAV are single-stranded DNA with the unique feature of crossing the blood–brain barrier (BBB) [27]. Herpes viruses also have unique features as they are double-stranded DNA viruses that permanently integrate into host cells and remain in latency in neuronal cells.

Viral vectors have been currently used in many clinical trials and have been proven to efficiently treat different neurological diseases, including childhood diseases such as lysosomal storage disorders (LSD), Duchenne’s muscular dystrophy, and spinal muscular atrophy (SMA). Lysosomal storage disorders are rare diseases with childhood onset characterized by loss of lysosomal functions and resulting in an accumulation of metabolites such as sphingolipids and mucoliposaccarids. Retrovirus- and AVV-mediated gene therapy for these metabolic disorders has been explored to restore the altered lysosomal enzymes [8,27,28,29]. Preclinical studies have successfully been performed in mice [30]. However, a major problem encountered with these gene transfer methods is their immunogenicity [27]. AAV gene therapy has also been extensively studied in SMA [4]. A single intravenous injection of an AAV-SMN transgene in mice was shown to fully restore SMN expression and completely rescue motor functions and prevent premature death [31]. When injected in neonatal mice, partial rescue of the pathological phenotype was observed and a time window for optimal administration to obtain complete rescue was suggested [32]. Expression of the transgene under the control of an opportunely introduced promoter or enhancer was demonstrated to enhance specificity and reduce toxicity [4]. Gene therapy for SMA is currently an approved treatment for SMA [33]. Recently, strategies to achieve specificity, to successfully cross the BBB, and to target specific brain regions included magnetic resonance imaging (MRI) delivery of viral vectors [34,35]. Even though gene therapy delivery with viral vectors has been shown to be efficient in several studies, the relatively small carrier capacity (especially of AAV), the non-desired insertional mutagenesis, and the unspecific targeting represent relevant drawbacks in their use [8,36,37,38,39]. Insertional mutagenesis has been proved by several studies to be the main challenge limiting the application of viral vectors for gene therapy delivery due to tumorigenesis as an important side effect [40,41]. Clinical studies showed the development of leukemia in patients treated with lentiviruses [41]. In fact, the solid risk of tumorigenesis linked to lentiviruses relies on integration with neighboring genes and affinity for oncogenes [41]. On the other hand, the second main challenge related to viral vectors is immunogenicity, especially when choosing AAV as gene delivery method [41,42]. Clinical trials reported a strong adaptive immune response against AAV’s capsids which produced neutralizing antibodies (NAb) as well as a strong innate T cell immune response [41]. Together, these drawbacks remarkably decrease the efficiency of viral vectors. Thus, scientists have switched their focus to alternative ways to deliver gene therapy such as non-viral vectors [40].

#### 2.1.2. Non-Viral Vectors: Cationic Polymers

Cationic polymers, due to their positive charge, can efficiently translocate into the nucleus and bind to DNA [43]. Cationic polymers include intelligent polymers and dendrimers. While dendrimers form covalent bonds and conjugate with high molecular weight molecules [44], intelligent polymers meet structural variations in response to stimuli that make them suitable to achieve stable and specific transgene expression [45]. When choosing cationic polymers to deliver therapeutic genes, molecules’ complexity and molecular weights needs to be accounted for both safety and efficiency [43]. Cationic polymers have been observed to be particularly useful for delivering siRNA in gene silencing approaches [46], reaching high silencing efficiency (up to 90%) [8,43]. Although the delivery of cationic polymers might sound attractive, their major disadvantage is toxicity [17], due to the DNA-polymer complex itself. Moreover, the dimension of the complex is not suitable for intravenous injections [47] and might lead to cytotoxic effects, including membrane disruption and loss of cell integrity [45].

#### 2.1.3. Lipid-Based Nanoparticles: Liposomes, Cationic Lipids and Ionizable Lipids

Liposomes are phospholipids molecules characterized by a water-based solution nucleus. When coated with cationic lipids, they efficiently bind to DNA [17]. Liposomes have an interesting property as their phospholipids coating can fuse with the cell membrane and release their content into the cell or be internalized via an endocytosis mechanism without eliciting an immune response [48]. Cationic lipids delivery of gene therapy has already been tested for central nervous system (CNS) neurological diseases [49]. Yet, when delivering gene therapy through liposomes, efficiency of this methods is low as most of them remain internalized into endosomes and further degrade via the lysosomal route. Advances in lipid-based nanoparticles technology ruled out these issues by introducing ionizable lipids [50]. Once internalized in endosomes, ionizable lipids are protonated and thus, not degraded [50]. Therefore, this class of molecules allow for efficient cytoplasmic release of genetic material and was demonstrated to have low toxicity [50].

### 2.2. Advanced Gene Therapy: Gene Therapy in the Future

Current advances in iPSCs methods combined with the CRISPR-Cas9 technology have triumphed over classical gene transfer methods (Table 1). Such advances have opened a new scenario to alternative methods of gene therapy in the near future: in fact, genes can be corrected or regulated in vitro, and consequently defective cells can be replaced in patients with engineered-corrected iPSCs. In the next section, we will discuss the most astonishing progress and we will describe how promising gene therapy using iPSCs in the future looks.

#### 2.2.1. Gene Editing–CRISPR-CAS9 Technology

In 2020, E. Doudna and J.A. Charpentier were awarded the Nobel prize for establishing clustered regularly interspaced palindromic repeats (CRISPR)-Cas9 technology as a gene editing tool able to revolutionize the genome engineering field. The CRISPR-Cas9 system originally developed in bacteria as a defense against viruses, a mechanism of adaptive immunity [51]. The main components of the CRISPR-Cas9 system are an RNA-guided Cas9 endonuclease and a single-guide RNA (sgRNA) [52]. The Cas9 protein is a nuclease that induces strand breaks in DNA. The guide RNA is a simplified combination of crRNA and tracrRNA that recruits Cas9 endonuclease to a specific target site [53]. The Cas9 nuclease and sgRNA form a Cas9 ribonucleoprotein (RNP), which binds and cleaves the specific DNA target [54] generating a double-stranded break (DSB), can be repaired by two endogenous self-repair mechanisms, the error-prone non-homologous end joining (NHEJ) pathway or the homology-directed repair (HDR) pathway [55]. These self-repair mechanisms are exploited to generate knock-out and knock-in, respectively.

When applied to the iPSCs, this methodology becomes particularly relevant. Patient-derived iPSCs can be gene edited to obtain isogenic controls, thus reducing the variability due to the genetic background. Before applying this technology, it was hard to discriminate between the effects of the mutations and the contribution of genetic background of cells. With the application of CRISPR/Cas9 technology, isogenic iPSCs can be generated, allowing to exclusively focus on the specific disease-causing mutation [56], and to demonstrate a rescue of the disease-related cellular endophenotype. Isogenic iPSCs obtained by mutation correction in genes have been generated to rescue the ALS phenotype [57]. In another study, mutated dystrophin was successfully corrected in an iPSC model of Duchenne muscular dystrophy [58]. Recently, the CRISPR-Cas9 technology has also been applied on an iPSC model of retinal degenerative diseases. Leber congenital amaurosis (LCA) is a rare childhood inherited retinal disease, with its more severe form caused by an intronic splice mutation in *CEP290* [59]. The successful removal of the mutation by CRISPR-Cas9 gene editing on patient-derived iPSCs has been obtained [60]. To date, the AAV-mediated gene therapy for this variant has been constrained by vector limited capacity that cannot carry the full-length cDNA. Hence, gene editing sounds as the most promising gene therapy approach [59].

CRISPR-Cas9 technology has already advanced the field of personalized medicine, as demonstrated by its application in hemoglobinopathies gene therapy. Beta-thalassemia (β-thal) and sickle cell disease (SCD), two of the most common genetic diseases, are caused by point mutations or small deletions in the *HBB* gene that affect mRNA transcription, splicing, or translation, which eventually lead to a deficiency in β-hemoglobin [61]. Recently, CRISPR-Cas9 technology in human progenitor cells (HSPCs) has been applied as a precise genome editing tool for treating β-thal and SCD. This approach provides the collection of CD34+ HSPCs from patients with Transfusion-dependent β-thal (TDT) and SCD and the destruction of *BCL11A* gene (B-cell lymphoma 11 A), a potent silencer of the fetal hemoglobin (HbF), resulting in reactivation of the γ-globin expression [62]. Fetal hemoglobin is a potent genetic modifier of the severity of β-thal and sickle cell anemia and differences in the levels of HbF that persist into adulthood affect the severity of sickle cell disease and β-thal [63]. CRISPR/Cas9-induced genome editing was performed in β-Thal iPSCs using piggyBac or a donor vector [64,65] or single strand oligodeoxynucleotides (ssODNs) as templates to generate point mutations and short sequence insertions in human cells and animal models [60,66,67]. The subsequent differentiation of β-Thal iPSCs into HSCs offered an opportunity for the autologous transplantation for disease treatment [68,69,70]. Nonetheless, off-targeting is a real drawback of the CRISPR-Cas9 technology. Thus, researchers have been making efforts to identify strategies to overcome this issue. Potential sites of off-target editing were identified by GUIDE-seq or computational methods and afterwards evaluated with the use of high coverage, hybrid-capture experiments by means of deep next-generation sequencing of edited CD34+ cells obtained from four healthy donors.

Transplantation therapy with CRISPR-Cas9 edited stem cells has started in 2019, when two international clinical trials were launched to treat young adults and adolescents with β-thal and SCD (NCT04208529, source: clinicalgov.com, accessed on 1 September 2021). Patients affected by TDT and SCD, respectively, received a single intravenous infusion of CTX001 (autologous CRISPR-Cas9– edited CD34+ HSPCs) and monitored for engraftment, adverse events, total hemoglobin, and hemoglobin fractions on high-performance liquid chromatography [62]. The trial is currently ongoing, and it involves 14 institutions in the USA, Canada, and Europe for the selection of patients, collection of cells to be “edited”, and the administration of the treatment. It is now clear that iPSCs can be engineered with the CRISPR-Cas9 technology, eventually overcoming the limitations of the classical methods in delivering gene therapy.

#### 2.2.2. Pros and Cons of CRISPR/Cas9 Gene Editing

The CRISPR/Cas9 gene editing is predicted to be a powerful tool in translational research thanks to its ease of use and cost-effectiveness. The probability that a CRISPR/Cas9 experiment is successful relies on well-designed single-guide RNA (sgRNA) and a lot of bioinformatic tools have been developed to assist researchers in the design of specific sgRNA.

The Zhang laboratory investigated sgRNA target specificity and they found that mismatch tolerance between SpCas9 complex and DNA is influenced by the number, position and distribution of mismatches. They have implemented a web-based software tool to facilitate sgRNA design and validation (http://crispr.mit.edu, accessed on 1 September 2021) based on a penalty matrix used to describe the effect of mismatch position. The penalty is between 0 and 1 where higher value means bigger effect on cleavage. Based on this penalty matrix, each sgRNA can be assigned a score according to its potential off-target sites, as evidence to choose appropriate sgRNAs. This method is widely used for sgRNA specificity score calculation, such as CRISPR-scan [71], CRISPR-DO [72], CHOPCHOP [73,74], and CRISPOR [75].

Despite these pros, the possibility to have off-target effects on the genome is real and to this aim many off-target detection tools have been developed.

In fact, off-targets remain the major imperfection of CRISPR/Cas9 technology. Since its development in 2014 [51], many researchers and companies have focused their attention towards the application of powerful methods to detect potential off-targets following CRISPR/Cas9 (Table 2).

In conclusion, CRISPR/Cas9 genome editing may lead to sequence mutation, deletion, rearrangement, oncogene activation and cell death, thus hindering the application of CRISPR/Cas9 system in research and clinics. In light of this, researchers are developing effective methods to detect the editing efficiency and off-target ratio willing to reduce off-target risk and improve the specificity of gene editing. These continuous efforts will most probably lead to improvement of the CRISPR/Cas9 gene editing technology in order to operate a system that can be applied to basic, but most importantly to translational applications [76,77,78].

### 2.3. The Future of Stem Cell Gene Therapy: Latest Evidence

As mentioned above, iPSCs embody an infinite source of patient-derived cells for modelling neurological diseases and for gene therapy correction and gene therapy drug screening. iPSCs can be derived from blood cells or fibroblasts and can be differentiated in motor neurons and retinal ganglion cells [79,80]. Evidence demonstrated how iPSCs can be efficiently manipulated with the latest genome editing technologies. Then, the engineered iPSCs can be transplanted into patients through cell therapy replacement. By using this approach, the first clinical trials have recently been approved for the treatment of several neurological disorders [80].

Though the major issue of cell therapy replacement is immune cellular compatibility, it has been knocked over in a recent study. By gene editing the human leukocyte antigens (HLA) genes, Xu and coworkers astonishingly attained engineered iPSCs immune-compatible with more than 90% of the world population [81]. HLA genes are part of the major histocompatibility complex (MHC), which accounts for cellular immunity response. These genes encode proteins exposing antigens on the cellular surface. There are two classes of HLA/MHC, class I genes expose antigens that recruits CD8^+^ and CD4^+^ lymphocytes, whilst class II expose antigens that cause T-helper to replicate and later recruit B lymphocytes. HLA loci vary amongst individuals, which accounts for the major cause of organ transplantation rejection and stem cell transplantation immune adverse reactions. In this study, two methods were used to manipulate HLA genes using the CRISPR-Cas9 technology, the generation of HLA class I homozygous iPSCs derived from HLA heterozygous donors and the depletion of HLA-A and HLA-B alleles obtaining HLA-C-only iPSCs [81]. Moreover, the MHC class II transactivator (CIITA) was also edited and iPSCs CIITA KO were generated [81]. The results proved that CRISPR-Cas9 engineered iPSCs HLA-C/CIITA retained suppressed the immune recruitment of cytotoxic CD8^+^ and CD4^+^ lymphocytes, thus, silencing the cytotoxic immune rejection response [81]. In allogeneic transplantation, these HLA-edited iPSCs could strongly expand donors’ compatibility [81].

Prospectively, this study represents the breakthrough in stem cell transplantation therapy being as the foundation for its application jointly with CRISPR-Cas9 gene editing. Indeed, making iPSCs immune-compatible thus opens a tangible future for cell transplantation therapy as immune rejection represents the major issue of feasibility of this gene therapy [81].

Another major hurdle of stem cell therapy is addressing the risk of tumorigenicity. The tumorigenicity potential of iPSCs when transplanted is related to three main factors: (i) non-complete terminal differentiation; (ii) non-complete silencing of pluripotency gene networks; (iii) reactivation of pluripotency genetic factors [82]. In the last few years, stem cell researchers have been focusing their efforts on discovering methods to control and minimize such risk. The breakthrough tackling this safety issue comes from a recent study which might have concrete clinical applications. Once more, by gene editing, Martin and coworkers created two orthogonal drug-inducible safeguard systems enabling to eliminate undifferentiated iPSCs in vitro. The safeguard systems developed by Martin et al. will possibly overcome safety hurdles facing stem cell transplantation therapy [83].

In addition, an increasing number of studies have been focusing on developing assays for screening tumorigenic mutations. These assays aim at identifying cancer-related mutations in the iPSC therapy product and consequently depleting them before transplanting. Evidence reported that methods such as FACS and qRT-PCR are effective in detecting undifferentiated iPSCs in vitro [84]. The identification of tumorigenic mutations in the iPSCs pool in vitro can also be performed by RNA-seq analysis [85]. Recently, another study has proposed the analysis of cellular senescence and the quantification of the minimum number of cells to be transplanted that does not cause teratoma formation as quality tests [86].

Following up these results, it is now clear that there is an emerging number of methods to check upon and control the tumorigenic potential of transplanted iPSCs.

In fact, preclinical and clinical studies on cell replacement, specifically in retinal degeneration diseases, have been already performed [80]. The first clinical study that transplanted iPSC-derived retinal pigmented epithelial cells for the treatment of macular degeneration diseases started in 2014 with a positive outcome in repairing patients’ vision [87]. Moreover, in 2018, the first clinical trial using stem cell therapy was approved for treating Parkinson’s disease [88]. Until now, the treatment for Parkinson’s disease aimed at temporarily restoring dopamine levels with scarce results. The main goal of stem cell therapy is instead to regenerate dopamine neurons as a permanent cure [89,90].

Japanese research stemming from Yamanaka’s work on iPSCs is on the cutting-edge of stem cell therapy research. A massive iPSC biobanking effort was established at CIRA (Center for iPS Cell Research and Application, Kyoto University, Japan). Similarly, other iPSC biobanks have been created worldwide, in the USA (CIRM, NYSCF and NIH) and Europe (HipSci in UK, EBiSC in UK and Germany, StemBANCC in France, Spain, Germany, UK, Sweden, Norway, Switzerland, Austria and Estonia).

### 2.4. Gene Therapy in the Clinics and Ethics

When translating gene therapy into the clinics, several key issues should be taken into consideration. The first relevant point is related to the selection of a proper route of administration. The choice is aimed at obtaining the maximum target specificity. With the classical methods of delivering gene therapy, some obstacles have been met. Firstly, off-target expression of therapeutic genes is relatively common and cytotoxicity in the surrounding tissues as well [17]. Secondly, classical gene therapy delivery methods can only be restricted to limited areas [17]. Finally, the brain is a complex structure and not easily accessible. Most of the therapeutics vectors employed cannot pass the BBB, and therefore targeting the central nervous system becomes problematic. Common routes of administration include intravenous and intramuscular delivery [4]. To achieve brain specificity, administration methods have recently been developed, such as intrathecal or intracerebroventricular delivery [4]. Parenchymal delivery of AAV vectors into brain structures has been improved by MR-guided inoculations and it is now being used in clinical trials [34]. Stem cell therapy looks upon these factors. Specificity is obtained by the selectivity of the cell type used and engineered stem cells can be infused intravenously with the advantage of a non-invasive therapeutic delivery.

Ethical concerns should also be considered. The safety of the therapy must be assessed, and adverse effects minimized to the greatest extent possible. Classical delivery methods showed some cytotoxic effects but were generally proven to be safe. On the other hand, stem cell therapy showed risk of tumorigenicity but was generally proven to be safe [87]. However, a strict monitoring of patients under treatment is required and upstream check of good manufactory practices (GMPs) of the therapeutics products is a must [87].

The results we discussed allow to consider that we are at the beginning of a new era in medicine, a new era for the treatment of neurological diseases.

## 3. Conclusions and Future Perspectives

**Figure 1:**
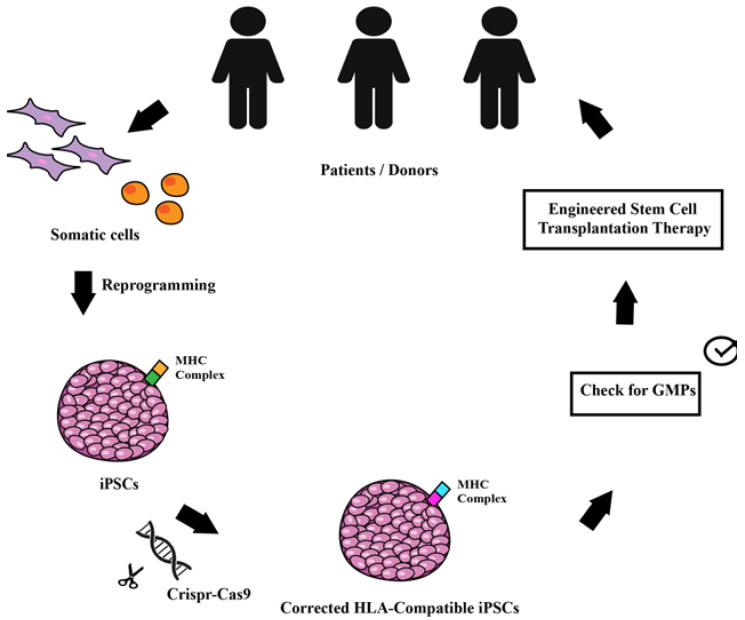
Schematic drawing of the workflow allowing the use of genetically modified iPSCs for transplantation therapy. Starting from the patients, somatic cells can be reprogrammed into iPSCs that are then corrected or modified with CRISPR/Cas9 gene editing with the aim to modify the MHC complex to make them compatible for a wider population of patients. The engineered stem cells can be used for autologous and/or for allogeneic transplantation and used as therapy for monogenic disorders.

Though gene therapy has been investigated for several decades, iPSCs technology applied using the latest gene editing methods (CRISPR-Cas9) represent the current emerging field. Huge steps forward have been made since the first employment of a therapeutic gene product. Even though proof of concepts for gene therapy vectors and preclinical studies are now established, benefits are not permanent, and efficiency is still low. The combined use of iPSCs and the CRISPR-Cas9 might represent a consolidated treatment for many neurogenetic disorders. Clinical trials with iPSCs have already started and the therapeutic application of stem cell therapy appears feasible by generating HLA-compatible iPSCs. These remarkable results represent the hope for a safe and truly efficient gene therapy (Figure 1). In the next decades, efforts will be focused on generating engineered donor-compatible iPSCs aimed at definitively reversing neurological disease phenotypes.

## Figures and Tables

**Table 1:** Advantages and disadvantages of classical and advanced gene therapy delivery methods.

Methods	Advantages	Disadvantages
Classical delivery methods:		
Viral vectors	Cell’s specificity, permanent integration, no pathogenicity, infection of quiescent cells	Insertional mutagenesis and consequent tumorigenic potential, immunogenic potential
Non-viral vectors	Efficient nucleus translocation, stable and specific gene expression	Cytotoxicity
Lipid-based nanoparticles	Cytoplasmic release with no immunogenic potential	Low efficiency
Advanced gene therapy	High efficient gene regulation and gene correction, immune-compatibility, upstream and downstream check and correction of tumorigenic iPSCs	High costs, long process, constant check of iPSCs gene therapy products and patients

**Table 2:** Methods for off-target detection (for further details see [76,77]).

Biased Methods	Unbiased Methods
Amplification of pre-selected off-target sites by PCR analysis followed by Sanger sequencing.	Whole genome sequencing
	Whole exome sequencing.
	ChIP-seq.
	GUIDE-seq.
	Bless.
	IDLVs.
	LAM-HTGTS.
	Digenome-seq.
	CIRCLE-seq.
	SITE-seq.
	GOTI.
	FISH
